# smdi: an R package to perform structural missing data investigations on partially observed confounders in real-world evidence studies

**DOI:** 10.1093/jamiaopen/ooae008

**Published:** 2024-01-31

**Authors:** Janick Weberpals, Sudha R Raman, Pamela A Shaw, Hana Lee, Bradley G Hammill, Sengwee Toh, John G Connolly, Kimberly J Dandreo, Fang Tian, Wei Liu, Jie Li, José J Hernández-Muñoz, Robert J Glynn, Rishi J Desai

**Affiliations:** Division of Pharmacoepidemiology and Pharmacoeconomics, Department of Medicine, Brigham and Women’s Hospital, Harvard Medical School, Boston, MA 02120, United States; Department of Population Health Sciences, Duke University School of Medicine, Durham, NC 27701, United States; Biostatistics Division, Kaiser Permanente Washington Health Research Institute, Seattle, WA 98101, United States; Office of Biostatistics, Center for Drug Evaluation and Research, United States Food and Drug Administration, Silver Spring, MD 20993, United States; Department of Population Health Sciences, Duke University School of Medicine, Durham, NC 27701, United States; Department of Population Medicine, Harvard Medical School and Harvard Pilgrim Health Care Institute, Boston, MA 02215, United States; Department of Population Medicine, Harvard Medical School and Harvard Pilgrim Health Care Institute, Boston, MA 02215, United States; Department of Population Medicine, Harvard Medical School and Harvard Pilgrim Health Care Institute, Boston, MA 02215, United States; Office of Surveillance and Epidemiology, Center for Drug Evaluation and Research, United States Food and Drug Administration, Silver Spring, MD 20993, United States; Office of Surveillance and Epidemiology, Center for Drug Evaluation and Research, United States Food and Drug Administration, Silver Spring, MD 20993, United States; Office of Surveillance and Epidemiology, Center for Drug Evaluation and Research, United States Food and Drug Administration, Silver Spring, MD 20993, United States; Office of Surveillance and Epidemiology, Center for Drug Evaluation and Research, United States Food and Drug Administration, Silver Spring, MD 20993, United States; Division of Pharmacoepidemiology and Pharmacoeconomics, Department of Medicine, Brigham and Women’s Hospital, Harvard Medical School, Boston, MA 02120, United States; Division of Pharmacoepidemiology and Pharmacoeconomics, Department of Medicine, Brigham and Women’s Hospital, Harvard Medical School, Boston, MA 02120, United States

**Keywords:** missing data, confounder, EHR, R, software, real-world evidence

## Abstract

**Objectives:**

Partially observed confounder data pose a major challenge in statistical analyses aimed to inform causal inference using electronic health records (EHRs). While analytic approaches such as imputation are available, assumptions on underlying missingness patterns and mechanisms must be verified. We aimed to develop a toolkit to streamline missing data diagnostics to guide choice of analytic approaches based on meeting necessary assumptions.

**Materials and methods:**

We developed the smdi (structural missing data investigations) R package based on results of a previous simulation study which considered structural assumptions of common missing data mechanisms in EHR.

**Results:**

smdi enables users to run principled missing data investigations on partially observed confounders and implement functions to visualize, describe, and infer potential missingness patterns and mechanisms based on observed data.

**Conclusions:**

The smdi R package is freely available on CRAN and can provide valuable insights into underlying missingness patterns and mechanisms and thereby help improve the robustness of real-world evidence studies.

## Background and significance

Administrative health insurance claims databases and electronic health records (EHRs) are important data sources to generate real-world evidence (RWE) when they are found fit-for-purpose for the study question at hand. While administrative health insurance claims databases have traditionally been the backbone for the majority of pharmacoepidemiologic studies, a notable drawback lies in their inability to capture important clinical prognostic factors like vital signs and labs. To overcome this limitation, substantial initiatives are underway, for instance in the FDA Sentinel initiative,^1^ linking claims databases and EHRs to generate real-world evidence (RWE) and complement data from randomized controlled trials (RCTs).^1^^,^^2^ Due to their capture of clinical details, EHR can significantly improve the ability to mitigate imbalances in prognostic factors between treatment groups.^3^ At the moment, substantial efforts focusing on the linkage of claims databases and EHR are underway, for instance, in the FDA Sentinel Initiative.^1^ However, prognostic factors coming from EHR are often only partially observed, posing a challenge to the statistical analysis and potentially leading to bias in treatment effect estimates if not handled appropriately.^4–6^

In order to inform decisions about the most appropriate analytic approach, it is useful to investigate the potential patterns and mechanisms that underlie the partially observed confounder (POC) data (see definitions box).^7–9^ Existing guidance frameworks have suggested various routine diagnostics to investigate missing data patterns and mechanisms. These methods comprise standard procedures such as comparing baseline characteristics and outcomes between patients with and without the POC,^10–14^ checking the ability to predict missingness^11^ and assessing if causal relationships between variables and their missingness are recoverable based on available data^15^ using directed acyclic graphs^16^^,^^17^ or M-graphs.^18^ However, these methods have so far only been described and tested in isolation from each other and no unified principled approach exists. In addition, the practical implementation of such diagnostics is time-consuming and consequently infrequently performed.^19–21^

**Table:** 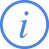
 Definitions: Basic missing data taxonomies.

Patterns (adapted from Van Buuren^7^) • Monotone pattern: If *Y_j_* is the *j*th column in a dataset *Y*, a missing data pattern is said to be *monotone* if the variables *Y_j_* can be ordered such that if *Y_j_* is missing then all variables *Y_k_* with *k > j* are also missing. This can occur, for example, in longitudinal studies with drop-out. • Non-monotone pattern: If the pattern is not monotone, it is called *non-monotone* or *general*. Mechanisms^11^ • Missing completely at random (MCAR): The missingness does not depend on any other observed or unobserved covariate(s). • Missing at random (MAR): The missingness depends and can be explained by other observed covariates. • Missing not at random (MNAR): The missingness depends on unobserved covariate(s). For example, the missingness may be explained by other covariate(s) which is/are not observed in the underlying dataset (MNAR_unmeasured_). The missingness can also just depend on the actual value of the partially observed covariate itself (MNAR_value_).

Considering these limitations, we have recently developed and evaluated a principled approach combining multiple missing data diagnostics^22^ using a database linkage from the Mass General Brigham Research Patient Data Registry EHR in Boston^23^ linked with Medicare fee-for-service claims data.^24^ The results of this large-scale study revealed that the combination of these diagnostics effectively identified underlying mechanisms and provided helpful guidance for the choice of appropriate analytic methods to handle POC data.

## Objective

To streamline the implementation of routine missing data diagnostics for POC data in RWE studies, we developed the smdi (structural missing data investigations) R package.^25^

## Methods

The smdi R package was written in R language (version 4.2.1). The package is available on the comprehensive R archive network (https://cran.r-project.org/web/packages/smdi) and GitLab (https://gitlab-scm.partners.org/janickweberpals/smdi) and can be installed via install.packages(“smdi”). To ensure quality, we implemented comprehensive unit tests with a coverage of 95.81% and established automated R CMD checks^26^ via continuous integration and deployment. Additional resources such as documentation and vignettes are provided on the package website: https://janickweberpals.gitlab-pages.partners.org/smdi.

## Results

### Main package functions

**Figure 1.**
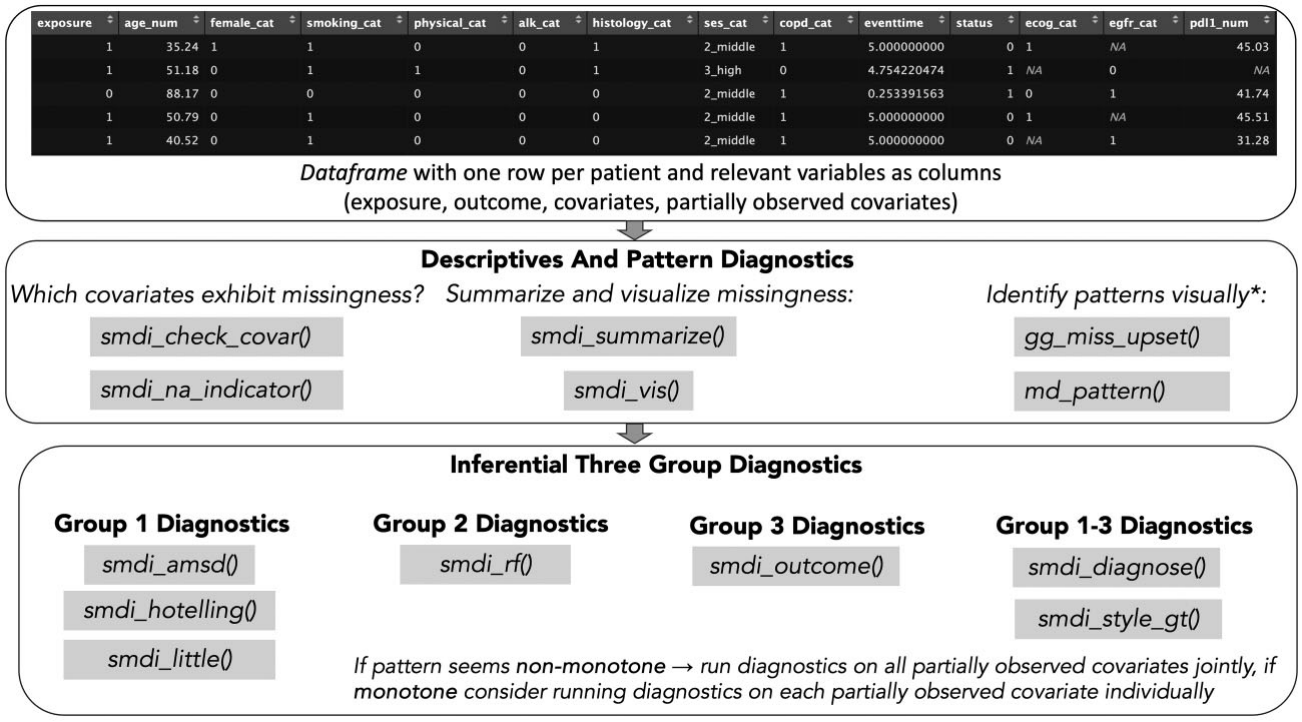
Overview of all smdi functions and suggested workflow to conduct missing data diagnostics. *gg_miss_upset() and md.pattern() are re-exports of the naniar and mice package, respectively.

Figure 1 illustrates the recommended workflow to systematically approach diagnostics on POCs.

The workflow is generally categorized into descriptives, pattern diagnostics, and inferential diagnostics on potentially underlying missingness mechanisms. In this section, we cover the principles behind the main package functions, a selection of parameters users can specify, the returned results and how these can be interpreted. Examples are illustrated using a synthetic dataset that is part of the package and simulates an oncology cohort with a binary exposure, a time-to-event outcome and several baseline confounders and prognostic covariates including 3 POCs (EGFR and PD-L1 [biomarkers] and ECOG [performance score]) following a MAR, MNAR, and MCAR mechanism, respectively (more details: https://janickweberpals.gitlab-pages.partners.org/smdi/articles/a_data_generation.html).

For all functions in the smdi package, a *dataframe* is expected (data parameter) as input with a format where one row represents one unique patient and the columns represent relevant variables, ie, exposure, outcome, fully observed covariates, and the POCs. Any non-informative columns, for example, patient identifiers, should be dropped from the dataframe before calling the functions. Throughout all functions, users have the option to specify a vector with the column name(s) of the POC(s) that should be investigated (covar parameter). If nothing is specified, all functions automatically consider any variable in the dataframe that exhibits at least one missing value.

Details on missingness assumptions, key statistical principles, and further information on all functions can be found in the Supplementary Methods and in the documentation of each respective function which can be accessed in R by preceding the function name with a question mark, eg:

? smdi_diagnose()

### Descriptives and pattern diagnostics

**Figure 2.**
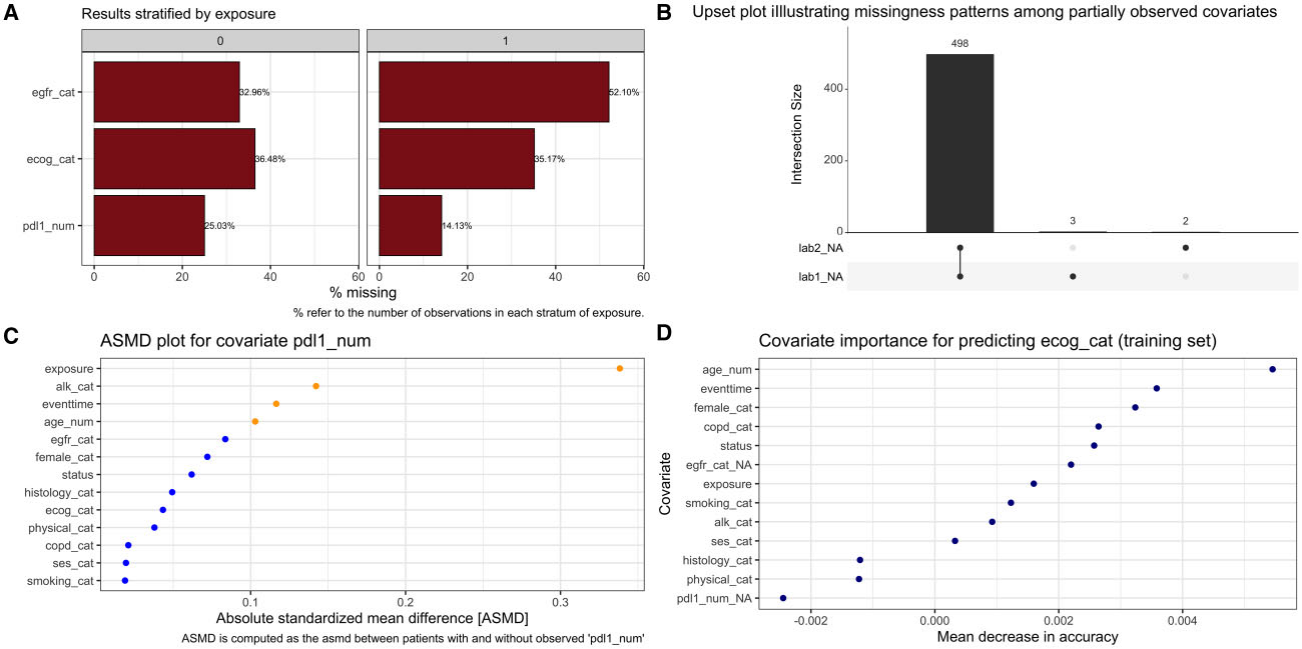
Exemplary visual outputs of the (A) smdi_vis(), (B) gg_miss_upset(), (C) smdi_asmd(), and (D) smdi_rf() functions, respectively. Sub-figure (A) displays the proportion of missing observations for each partially observed covariate stratified by exposure. The upset plot in sub-figure (B) demonstrates how a monotone missingness pattern between partially observed covariates can be visually inspected using a set visualization technique.^28^ Sub-figure (C) illustrates absolute standardized mean differences (ASMDs) in patient characteristics between patients with and without a value observed for the PD-L1 (pdl1_num) biomarker as a measure of imbalance. Sub-figure (D) demonstrates the variable importance of fully observed covariates for predicting missingness in the partially observed ECOG performance score variable (ecog_cat).

As a first step to explore the missingness in new datasets, the smdi package provides a few basic functions to describe and summarize missingness across all covariates. The smdi_summarize() function returns the amount and proportion of missing observations, which can also be stratified by a grouping variable. The smdi_vis() function returns a corresponding bar chart plot (example Figure 2A).

To visually inspect potential missing data patterns, we re-exported the gg_miss_upset() function of the naniar package.^27^ This function uses a set visualization technique to visually infer potential (non-)monotone patterns based on the number of intersecting missing observations across all POCs.^28^ For example, a monotone pattern could be visually evident if, for a set of 2 or more lab variables which are typically measured together as part of a lab panel (eg, renal or liver panel), the missingness of one lab is indicative of the missingness in the other lab and hence all or the majority of combinations of cells are missing together (example Figure 2B). The md.pattern() function, a re-export of the mice package,^29^ provides a similar functionality and returns a matrix displaying the frequency of each observed missing data pattern.

### Inferential three group diagnostics

The core functions to infer potentially underlying missingness mechanisms are categorized into 3 group diagnostics based on their general analytic properties (Table 1).

**Table 1.** Overview of the main functions in smdi to characterize potential underlying missingness mechanisms.

Function	Description	Generic S3 print() output	Object output	Interpretation
Group 1 Diagnostics—Comparing the distribution of observed covariates between patients with versus without a value for the partially observed covariate				
smdi_asmd()	Computes the absolute standardized mean differences (ASMDs) of patient characteristics between patients with versus without a value for the partially observed covariate(s)	Aggregated summary table of the average/median and minimum/maximum ASMD range for all specified partially observed covariates	- Detailed Table 1 illustrating distributions and individual ASMD for each compared patient characteristic - ggplot2 graph illustrating the individual ASMD for each compared patient characteristic in descending order - Aggregate summary of the average/median and minimum/maximum ASMD range for the selected partially observed covariate	- ASMD <0.1: no imbalances in observed patient characteristics; missingness may be likely completely at random or not at random (∼MCAR, ∼MNAR) - ASMD >0.1: imbalances in observed patient characteristics; missingness may be likely at random (∼MAR)
smdi_hotelling()	Computes Hotelling’s multivariate *t*-test for each partially observed covariate, examining patient differences conditional on having an observed covariate value or not.	Aggregated summary table of the Hotelling’s test *P*-values for all specified partially observed covariates	Detailed Hotelling test statistics	High test statistics and low *P*-values indicate differences in baseline covariate distributions and null hypothesis would be rejected (∼MAR)
smdi_little()	Computes a single global chi-square test statistic across all partially observed covariates with a null hypothesis that the data are missing completely at random.	Detailed Little’s test statistics	Detailed Little’s test statistics	High test statistics and low *P*-values indicate differences in baseline covariate distributions and null hypothesis would be rejected (∼MAR)
Group 2 Diagnostics—Assessing the ability to predict missingness based on observed covariates				
smdi_rf()	Trains and fits a random forest classification model to assess the ability to predict missingness indicator for the partially observed covariate(s).	Aggregated summary table with the area under the receiver operating characteristic curve (AUC) value for each partially observed covariate	- Individual AUC value - ggplot2 figure illustrating the variable importance for the prediction made expressed by the mean decrease in accuracy per predictor - Estimated out-of-bag (OOB) error	- AUC values ∼ 0.5 indicate completely random or not at random prediction (∼MCAR, ∼MNAR) - Values meaningfully above 0.5 indicate stronger relationships between covariates and missingness (∼MAR)
Group 3 Diagnostics—Evaluates whether missingness of a covariate is associated with the outcome				
smdi_outcome()	Fits outcome model (linear, glm, or proportional hazards depending on the outcome under study) with the missingness indicator of the partially observed covariate(s). The estimates are computed both as a univariate model (just considering the missingness indicator) and an adjusted model with all covariates in the dataset.	Aggregated summary table with the univariate and adjusted estimate for each partially observed covariate	Aggregated summary table with the univariate and adjusted estimate for each partially observed covariate	- No association in either univariate or adjusted model and no meaningful difference in the log HR after full adjustment (∼MCAR). - Association in univariate but not fully adjusted model (∼MAR). - Meaningful difference in the log HR also after full adjustment (∼MNAR).

#### Group 1 diagnostics

The aim of the smdi_asmd(), smdi_hotelling(), and smdi_little() functions is to explore dissimilarities in patient characteristics between those with and without observed values for the POC. According to Rubin’s framework,^8^ when missingness is at random (MAR), it can be explained by observed covariates. Consequently, significant differences in patient characteristics would be expected under a MAR mechanism between strata of patients with and without the POC. If the missingness depends only on unobserved factors (missing not at random [MNAR]) or does not depend on either observed or unobserved covariates (missing completely at random [MCAR]), differences should not be observable.

To quantify such differences, the smdi_asmd() function computes absolute standardized mean differences (ASMDs) of observed patient characteristics.^30–32^ The function returns an *asmd* object which displays an aggregated summary of the average or median ASMD along with a corresponding range of minimum and maximum ASMDs for each POC, respectively. The object also returns detailed “ Table 1’s” and plots^33^ for each POC displaying the distributions of observed covariates and resulting ASMDs between patients with and without an observed value for the POC (example Figure 2C).

The smdi_hotelling() and smdi_little() functions complement the smdi_asmd() function by examining differences in patient characteristics as a formal statistical hypothesis test. Hotelling’s test^12^^,^^34^ formalizes this as a multivariate *t*-test, which means that smdi_hotelling() returns a test statistic and *P*-value for each POC. In contrast, smdi_little()^13^^,^^27^ computes a single global chi-square test statistic and *P*-value across all POCs with the null hypothesis that the data are (globally) MCAR.

**Figure 3.**
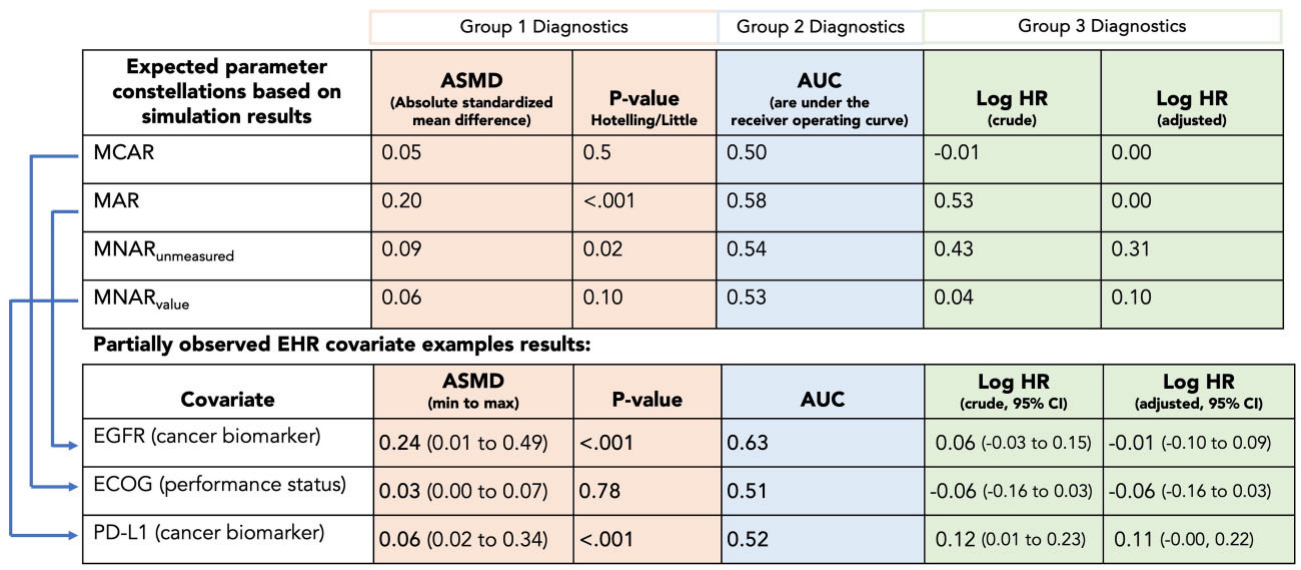
Example of how smdi diagnostics can be applied to compute and compare diagnostic parameters of partially observed covariates to expected parameters of common missingness mechanisms based on a former large-scale simulation study.^22^

Applying group 1 diagnostics to the synthetic example dataset would indicate that the ECOG POC (median ASMD 0.03, min-max 0.00-0.07, *P*-value .78) does not show any differences in observed patient characteristics between patients with and without and observed value for ECOG which would give evidence for a MCAR mechanism (Figure 3 bottom, Group 1 diagnostics—orange boxes). Conversely, in the case of EGFR and PD-L1, the group 1 diagnostics display larger differences and hence may rather underlie a MAR or MNAR mechanism (Figures 2C and 3).

#### Group 2 diagnostics

Group 2 diagnostics assess the ability to predict missingness based on observed covariates via the smdi_rf() function. This function trains and fits a random forest classification model^11^^,^^35^ to predict the missing indicator of each POC given exposure, outcome, follow-up time, and covariates plus missingness indicator for other POC as the predictors. If the resulting area under the receiver operating characteristic curve (AUC) is meaningfully >0.5, this would give some evidence for MAR/against MCAR being the underlying missingness mechanism. In case of values close 0.5, this would indicate the model is unable to discriminate missing versus observed values based on available data; this could be due to a mechanism that is close to MCAR or one where the missingness is associated with unmeasured data (MNAR).

The function returns an object of class *rf* which generically prints an overview of the AUC value of each POC. The AUC is based on the prediction made in the respective test dataset which is sampled as part of the function and for which the train-test split ratio, number of trees, and CPU cores to parallelize over can be specified (train_test_ratio, ntree, and n_cores parameter, respectively).^35^^,^^36^ The *rf* object further returns a graph for each POC displaying the relative importance of the predictors in the training dataset expressed as the mean decrease in accuracy (example Figure 2D). This metric can be valuable for interpreting and identifying strong predictors of missingness. It quantifies how much the accuracy of the prediction (ie, the ratio of correct predictions to the total number of predictions made) would decrease if we excluded a specific predictor from the model. In case of inflated AUC values (>0.9), the function prompts a message to the user reporting the most important predictor. If in such a scenario missingness in another POC is identified as a perfect predictor, the presence of a monotone missing data pattern may be likely in which case it is recommended to run the diagnostics for each POC independently rather than jointly.

Figure 3 (Group 2 diagnostics—blue boxes), for example, illustrates the AUC values of the output of smdi_rf() when applied to the synthetic example dataset. Since the missingness of the EGFR POC follows a true MAR mechanism, the resulting AUC of 0.63 is expectedly meaningfully higher than what is observed for ECOG (0.51) and PD-L1 (0.52) which follow a true MCAR and MNAR mechanism, respectively.

#### Group 3 diagnostics

The third group of diagnostics with the smdi_outcome() function examines the association of the missingness indicator of the POC and the outcome under study. The function computes both a univariate model and a model adjusted for all other covariates in the dataset. In simulations, we discerned distinct patterns in both univariate and adjusted associations between the missing indicator and the outcome, closely mirroring simulated missingness mechanisms (Figure 3, top).^22^ As expected, under a MCAR mechanism the simulation suggested no difference in the outcome between patients with and without a value for the POC. Under MAR, given that missingness can be sufficiently explained by observed covariates, a spurious association in the univariate model disappeared after adjustment. If the missingness followed any MNAR mechanism, an association was observed regardless of adjustment.

smdi_outcome() supports multiple outcome regression types including linear regression (*lm*^37^) for continuous outcomes, Cox proportional hazards model (*coxph*^38^) for time-to-event outcomes, and generalized linear regression models (*glm*^37^) for which the family of conditional distributions of the outcome can be selected using the glm_family parameter (the default is binomial(link="logit")). Besides the regression type (model parameter) and the glm_family (in case of a glm model), users need to specify the column containing the outcome using the form_lhs parameter (eg, Surv(eventtime, status) in case of a Cox model). The function returns a table with univariate and adjusted beta coefficients and 95% CIs for each POC.

Demonstrating the utilization of smdi_outcome() using the example dataset, the derived logHR coefficients for the missingness indicators of the POCs (Figure 3, bottom, Group 3 diagnostics—green boxes) align with the anticipated outcomes from our simulations.^22^ Specifically, EGFR manifests no discernible difference in the outcome after adjustment for fully observed covariates (logHR −0.01, 95% CI, −0.10 to 0.09), suggesting a MAR mechanism. ECOG exhibits no distinction in either the unadjusted or adjusted model (logHR −0.06, −0.16 to 0.03), indicating MCAR. Conversely, PD-L1 showcases differences in the outcome in both models, suggesting an MNAR context.

*smdi_diagnose() to compute all three group diagnostics*

Finally, the smdi_diagnose() function enables users to compute all of the above-discussed group diagnostics within a single function call.

# minimal example of a smdi_diagnose() function call

 smdi_diagnose(

 data =smdi_data,

 model =“cox”,

 form_lhs =“Surv(eventtime, status)”,

 n_cores = 3

 )

The function returns an object of class *smdi* containing a table with the results of all diagnostics for each specified POC and Little’s test *P*-value across all covariates (Table 2). By cross-checking all resulting diagnostic parameters to expected estimates as illustrated in in the above examples (Figure 3),^22^ the diagnostics can provide valuable insights into underlying missingness mechanisms and thereby help elucidate if analytic approaches such as imputation analyses are viable options.

**Table 2.** Example output of the smdi_diagnose() function applied to the examplary smdi_data dataset.

Covariate	ASMD (min/max)^a^	*P* Hotelling^a^	AUC^b^	Beta univariate (95% CI)^c^	beta (95% CI)^c^
ecog_cat	0.029 (0.003, 0.071)	.783	0.510	−0.06 (−0.16 to 0.03)	−0.06 (−0.16 to 0.03)
egfr_cat	0.243 (0.010, 0.485)	<.001	0.629	0.06 (−0.03 to 0.15)	−0.01 (−0.10 to 0.09)
pdl1_num	0.062 (0.019, 0.338)	<.001	0.516	0.12 (0.01-0.23)	0.11 (−0.00 to 0.22)

In this example, ECOG performance score (ecog_cat) shows no imbalances in patient characteristics between patient with and without an observed value (absolute standardized mean difference [ASMD] 0.029, *P*[Hotelling] .783, group 1 diagnostic). Additionally missingness cannot be predicted well (AUC = 0. 510, group 2 diagnostic) and no difference in the outcome can be observed between patients with and without ecog_cat (log HR −0.06 [95% CI, −0.16 to 0.03], group 3 diagnostic). Accordingly, the missingness diagnostics indicate that ECOG follows a missing completely at random missingness (MCAR) mechanism. Similarly, the EGFR (egfr_cat) and PD-L1 (pdl1_num) biomarker variables can be interpreted as following a missing at random (MAR) and missing not at random value (MNARvalue) mechanism. See also Figure 3. *P* little: <.001.

Abbreviations: ASMD, median absolute standardized mean difference across all covariates; AUC, area under the curve; beta, beta coefficient; CI, confidence interval; max, maximum; min, minimum.

a Group 1 diagnostic: Differences in patient characteristics between patients with and without covariate.

b Group 2 diagnostic: Ability to predict missingness.

c Group 3 diagnostic: Assessment if missingness is associated with the outcome (univariate, adjusted).

The smdi_style_gt() function is an ancillary function that takes an object of class smdi and produces a formatted and publication-ready gt table^39^ which can be seamlessly exported to different file formats (eg, .docx, .pdf, etc.) for reports or manuscripts.

## Discussion

Missing data are ubiquitous in RWE studies involving EHR and may introduce bias if not handled appropriately. To address this issue, we developed the smdi R package to streamline routine diagnostics of missing data.

The package should be used with certain limitations in mind. Most importantly, the true underlying mechanism causing the missing data can never be inferred with absolute certainty from the observed data. Therefore, it is important to complement diagnostic results with substantive expert knowledge to factor in how covariates are measured in routine care, which could be system-specific, and contextualize potential reasons for missingness. This collaborative approach allows for a contextualized understanding of potential causes for missing data in EHR.

## Conclusions

The smdi R package is a powerful and convenient tool to implement principled routine missing data diagnostics in RWE studies. This will improve the robustness of studies involving POCs by elucidating if certain analytic approaches are viable for a given dataset.

## Supplementary Material

ooae008_Supplementary_Data

## Data Availability

The R package presented in this study and corresponding data can be downloaded from the comprehensive R archive network (CRAN) via install.packages(“smdi”) (version 0.2.2 at time of manuscript submission) or from https://janickweberpals.gitlab-pages.partners.org/smdi. This manuscript was written using Quarto version 1.3.433 (https://quarto.org/) and R version 4.1.2. All R code, materials, and dependencies can be found at https://gitlab-scm.partners.org/drugepi/smdi-manuscript or https://github.com/janickweberpals/smdi-manuscript.
