# Supplementary material for: smdi: an R package to perform structural missing data investigations on partially observed confounders in real-world evidence studies
Source: JAMIA Open. 2024 Jan 31;7(1):ooae008. doi: 10.1093/jamiaopen/ooae008 (PMC10833461; doi:10.1093/jamiaopen/ooae008)
Supplement: ooae008_Supplementary_Data [file ooae008_supplementary_data.pdf]

Supplementary Material

smdi: An R package to perform structural missing data investigations on partially observed confounders in real-world evidence studies

Authors: Janick Weberpals[1], RPh, PhD, Sudha R. Raman[2], PhD, Pamela A. Shaw[3], PhD, MS, Hana Lee[4], PhD, Bradley G. Hammill[2], DrPH, Sengwee Toh[5], ScD, John G. Connolly[5], ScD, Kimberly J. Dandreo[5], MS, Fang Tian[6], PhD, Wei Liu[6], PhD, Jie Li[6], PhD, José J. Hernández-Muñoz[6], PhD, Robert J. Glynn[1], PhD, ScD, Rishi J. Desai[1], PhD

Author affiliations:

[1] Division of Pharmacoepidemiology and Pharmacoeconomics, Department of Medicine, Brigham and Women's Hospital, Harvard Medical School, Boston, MA

[2] Department of Population Health Sciences, Duke University School of Medicine, Durham, NC

[3] Biostatistics Division, Kaiser Permanente Washington Health Research Institute, Seattle, WA

[4] Office of Biostatistics, Center for Drug Evaluation and Research, US Food and Drug Administration, Silver Spring, MD

[5] Department of Population Medicine, Harvard Medical School and Harvard Pilgrim Health Care Institute, Boston, MA

[6] Office of Surveillance and Epidemiology, Center for Drug Evaluation and Research, US Food and Drug Administration, Silver Spring, MD

1 Supplementary Methods

1.1 Missingness assumptions

For the design and validation of the `smdi` R package functions, we employed comprehensive simulations and a real-world example [1]. These simulations followed structural assumptions on realistic missingness generating mechanisms that could be expected in electronic health record (EHR) data. The below directed acyclic diagrams (DAG) [2,3], where a directed edge represents a causal effect of one variable on another variable, outline and illustrate these assumptions along with a brief explanations and exemplary real-world EHR scenarios. Since the `smdi` package was developed with focus on partially observed confounders (as opposed to missingness in exposure or outcome), we simplified the below DAGs by leaving out the nodes for exposure and outcome for improved readability.

Notation: C = Fully observed confounder, POC = True values of the partially observed confounder, POCobs = Observed portion of the partially observed confounder, M = Missingness of POC with M=0 fully observed and M=1 fully missing, U = Unobserved confounder.

1.1.1 Missing completely at random (MCAR)

The missingness (M) of the partially observed confounder (POC) is independent of any observed and unobserved confounders. That is, no edge directs to M and the missingness is completely at random (MCAR).

Real-world example: A machine breaks that is used to measure and analyze a certain biomarker.

Supplementary Figure 1: Directed acyclic graph displaying a missing completely at random (MCAR) scenario.

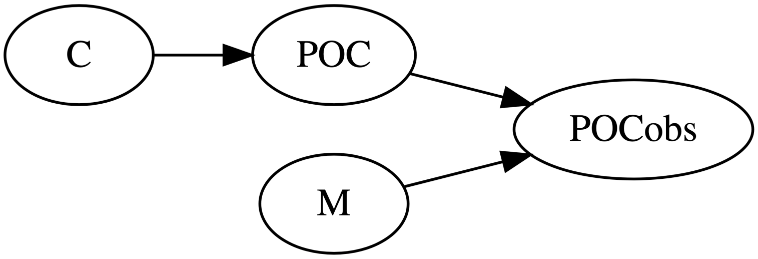

1.1.2 Missing at random (MAR)

The missingness (M) of the partially observed confounder (POC) can be explained by one or multiple fully observed confounders (C). That is, there is an edge from C to M and the missingness is at random (MAR).

Real-world example: Older patients are more likely to receive a certain biomarker test and age is a fully observed confounder which is measured for every patient in the dataset.

Supplementary Figure 2: Directed acyclic graph displaying a missing completely at random scenario.

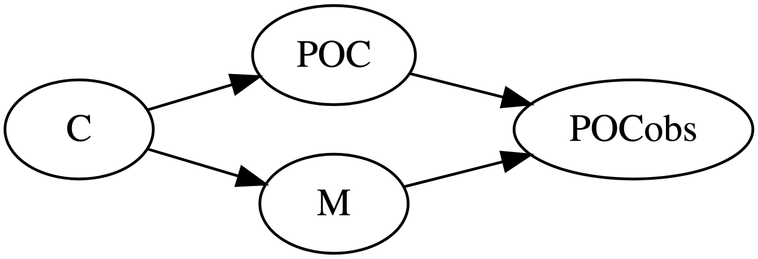

1.1.3 Missing not at random - unmeasured (MNAR$_{unmeasured}$)

The missingness (M) of the partially observed confounder (POC) can only be explained by one or multiple confounder(s) which are not observed in the dataset.

Real-world example: Patients with a certain performance status, which is not observed in the dataset, are more likely to receive a lab test.

Supplementary Figure 3: Directed acyclic graph displaying a missing not at random (unmeasured) scenario.

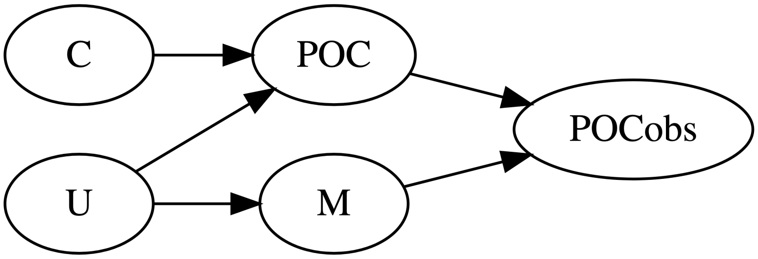

1.1.4 Missing not at random - value ($MNAR_{value}$)

The missingness (M) of the partially observed confounder (POC) depends on the true value of the partially observed confounder itself.

Real-world example: Patients with a history of normal biomarker values are systematically less likely to be tested again for the same biomarker.

Supplementary Figure 4: Directed acyclic graph displaying a missing not at random (value) scenario.

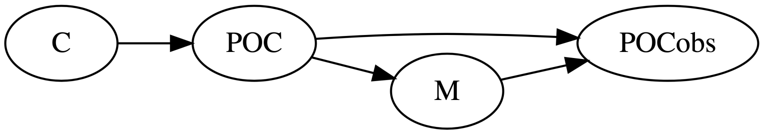

1.2 Missingness characterization

This section provides extensive information and details on key statistical principles and methods employed in the `smdi` package. Details on all package functions can also be found in the corresponding documentation section of the `smdi` package website or accessed in `R` by executing the function name preceded with a question mark, e.g.:

```
?smdi_diagnose()
```

A comprehensive illustration of an exemplary workflow of the `smdi` package can be found in the vignette `Routine structural missing data diagnostics` on the package website.

1.2.1 Utility functions

The `smdi` package comes with a few in-built utility functions which are called in the background by other `smdi` functions, but which can also be used as standalone functions by users if desired.

`smdi_check_covar()` checks for any partially observed covariate in a given dataset (specified by the `data` parameter) and returns a vector with the names of covariates exhibiting missingness. With the `covar` parameter, users can specify any specific covariates(s) of interest or, in case this parameter is not specified, any covariate with at least one missing value is returned. The function also provides further logical checks, e.g., if all covariates specified in `covar` are actually present in the dataset and if all ovariates specified in `covar` are truly missing (as sometimes missingness is explicitly coded as a “Missing” level or similar instead of `NA`).

smdi_na_indicator(): This function takes a dataset (data parameter) and creates binary missing indicator variables for covariates with at least one missing value. If covar is not specified, all possible variables in the dataset are considered and if covar is specified with covariates(s) of interest, only those are considered. With the logical drop_NA_col parameter, users can decide if the original covar variables should be dropped or retained in the dataset in addition to the created binary missing indicator variables.

smdi_style_gt() takes an object of class smdi (i.e., the output of the smdi_diagnose() function which returns all three group diagnostics) and formats the table as a publication-ready gt() table object [4] which can be exported to any format that is supported by gt::gtsave() including .pdf or .docx files.

1.2.2 Descriptives

smdi_summarize() and smdi_vis() compute and visualize the number and proportion of missing values for every covariate specified with covar. The output is a simple table (smdi_summarize()) or a vertical bar chart plot (ggplot2 object [5], smdi_vis()). Optionally, results can also be returned stratified by another variable which can be specified using the strata parameter.

gg_miss_upset() and md.pattern() are re-exports of the naniar [6] and mice [7] packages, respectively, both of which are useful functions to inspect missing data patterns. The gg_miss_upset() achieves this by using set visualization techniques as described in detail by Ruddle et al. [8]. In brief, the function returns a bar chart (*Upset plot*) that visualizes the number of intersecting missing observations across all POCs. It can be thought of as a high-dimensional Venn diagram for which, in case of monotone patterns, all or the majority of combinations of cells are missing simultaneously. The md.pattern() function has a similar functionality and returns a matrix in which each row corresponds to an observed missing data pattern. The rows and columns of the matrix are sorted in increasing amounts of missing information and thereby give an overview on how frequent a certain missing data pattern is.

1.2.3 Group 1 diagnostics

The featured group 1 diagnostics in smdi focus on investigating potential differences in the distribution of characteristics between patients with and without an observed value for a POC. To that end, different analytic approaches are leveraged which are described in detail for each function below.

smdi_hotelling(): This function computes a two-sample Hotelling's T-squared test for the difference in two multivariate means based on the Hotelling's T-Square test statistic [9,10]. It assesses whether there is a statistically significant difference between the means of two groups in multivariate data. This test is an extension of the univariate t-test that examines for two groups (i.e., patients with and without a value observed for the confounder of interest) with p

variables (e.g., patient or disease characteristics) whether their mean vectors (p-dimensional vectors of means) are significantly different. To that end, the T^2 statistic is derived by a vector of covariate means (i.e, one element for each covariate) [11], which are represented as $\bar{x}_1$ (vector of covariate means in group 1) and $\bar{x}_2$ (vector of covariate means in group 2) in Equation 1 below (with n_1 and n_2 representing the sample sizes of group 1 and 2, respectively, and S_p representing the pooled variance-covariance matrix) (Equation 1 adapted from [12]). To implement Hotelling's test in the `smdi` package, we built a wrapper around the `Hotelling::hotelling.test()` function [10].

$$T^2 = (\bar{\mathbf{x}}_1 - \bar{\mathbf{x}}_2)^T \{\mathbf{S}_p(\frac{1}{n_1} + \frac{1}{n_2})\}^{-1}(\bar{\mathbf{x}}_1 - \bar{\mathbf{x}}_2) \tag{1}$$

`smdi_little()`: Little's test, as opposed to Hotelling's test, performs a single global test to evaluate the missing completely at random (MCAR) assumption [13]. It was developed since an approach like Hotelling's test can raise concerns regarding multiplicity due to the multiple testing of each variable. Little's test first identifies a set of subgroups that share the same missing data patterns j. Across these missing data patterns (n_j), it then tests for mean differences on every variable by comparing the observed means ($\hat{\mu}_j$) versus expected population means ($\hat{\mu}j^{(\mathrm{ML})}$) which are estimated using a maximum likelihood (ML) expectation-maximization (EM) algorithm as implemented by the `norm::prelim.norm()` and `norm::em.norm()` functions in `R` (Equation 2 adapted from [14]). Under the assumption of MCAR, the test statistic (d^2) approximates a chi-square distribution and is derived by computing the sum of squared standardized differences between the subgroup means and the expected population means weighted by both the estimated variance-covariance matrix (where $\hat{\Sigma}_j$ is the maximum likelihood estimate of the covariance matrix) and the respective subgroup sizes [14,15]. To implement Little's test in the `smdi` package, we built a wrapper around the `naniar::mcar_test()` function [6].

$$d^2 = \sum_{j=1}^{J} n_j(\hat{\mu}_j - \hat{\mu}j^{(\mathrm{ML})})^T \hat{\Sigma}_j^{-1}(\hat{\mu}_j - \hat{\mu}j^{(\mathrm{ML})}) \tag{2}$$

For both `smdi_hotelling()` and `smdi_little()`, a high test statistic and a low p-value would indicate differences in the groups compared and reject the null hypothesis that the missingness follows a MCAR mechanism. A limitation of both Hotelling's and Little's test is that they assume continuous variables following multivariate normality. Real-world data, however, are often of binary nature and to consider categorical data in the computation of these tests, categorical data are one-hot encoded to binary dummy variables before performing each test. In addition, these tests are sensitive to just small differences when study sizes are large which is why we recommend to perform these tests jointly with `smdi_asmd`.

`smdi_asmd()`: The `smdi_asmd` function computes the absolute standardized mean difference (ASMD) as a statistical measure for assessing dissimilarities in disease and patient characteristics between individuals with and without observed value for a specific POC of interest. If the

median/average ASMD is high, this may indicate imbalance in patient covariate distributions which may be indicative of the POC following a missing at random (MAR) mechanims, i.e. the missingness is explainable by other observed covariates. Similarly, no imbalance between observed covariates may be indicative that missingness cannot be explained with observed covariates and the underlying missingness mechanism may be completely at random (MCAR) or not at random (e.g., missingness is only associated with unobserved factors or through the POC itself). This methodological approach follows the same theory that is often applied to measure covariate balance between two groups before and after matching or weighting on propensity scores, which are balancing scores often used to reduce confounding in real-world evidence studies [16]. While there isn't a universally established standard for the threshold of the ASMD indicating significant imbalance, an ASMD below 0.1 is often considered indicative of a negligible difference in the mean or prevalence of a covariate between two groups [16,17]. The advantage of ASMDs to assess the balance of patient characteristics between two groups with and without an observed value for the POC is that they are not influenced by sample size, do not come with many assumptions, are easy to interpret and applicable to various types of covariates. Equation 3 and Equation 4 illustrate how ASMDs are computed for continous and binary covariates with $\bar{x}$, s^2 and $\hat{p}$ indicating the mean, sample variance and prevalence of the binary variable of the covariate in patients with a missing (M) and an observed (O) value for the POC, respectively. In the `smdi` package, ASMDs are extracted from tableone objects using the `ExtractSmd` function [18].

$$ASMD_{continous} = |\frac{(\bar{x}_M - \bar{x}_O)}{\sqrt{\frac{s_M^2 + s_O^2}{2}}}| \tag{3}$$

$$ASMD_{binary} = |\frac{(\hat{p_M} - \hat{p_O})}{\sqrt{\frac{\hat{p_M}(1-\hat{p_M}) + \hat{p_O}(1-\hat{p_O})}{2}}}| \tag{4}$$

1.2.4 Group 2 diagnostics

`smdi_rf()`: The Group 2 diagnostics, implemented in the `smdi_rf()` function, assesses the ability to predict missingness based on observed covariates via the `smdi_rf()` function. This function trains and fits a random forest classification model [3,19] to predict the missing indicator of each POC given exposure, outcome, and covariates plus missingness indicator for other POC as the predictors. We chose a random forest classification model for the purpose of this package due its many beneficial features within the context of data structures that are frequently encountered in routine healthcare databases:

- Ability to implicitly model nonlinear and non-additive relationships between observed variables (i.e., higher order terms do not need to be explicitly specified).

- Recursive partitioning models like random forests have been found to work particularly well with sparse tabular data, which is the typical data type that is used for real-world evidence studies [20,21].
- Random Forests provide transparent feature importance measures, indicating the variables contributing significantly to predicting missingness. This aids in identifying key features driving the missingness mechanisms in the dataset.
- Multiple other studies have previously reported good result of this random forest-based approach for the purpose of diagnosing missing data [3,22].

The default training parameters of the random forest model include a train-test split of the data (default if 70% training and 30% testing), which can be changed using the `train_test_ratio` parameter, and the number of trees to grow using `ntree` (default is 1000 decision trees since a higher number of trees typically give more stable results). Users can optionally select `tune = TRUE` which will perform a 5-fold cross validation and a random search for the optimal number of variables randomly sampled as candidates at each split (`mtry`). However, users should be aware that this may lead to longer computation times with larger datasets which is why the default is set to `FALSE`. In summary, the following parameters are part of this function:

- `data`: dataframe or tibble object with partially observed/missing variables
- `covar`: character covariate or covariate vector with partially observed variable/column name(s) to investigate. If NULL, the function automatically includes all columns with at least one missing observation and all remaining covariates will be used as predictors
- `train_test_ratio`: numeric vector to indicate the test/train split ratio, e.g. c(.7, .3) which is the default
- `tune`: if TRUE, a 5-fold cross validation is performed combined with a random search for the optimal number of optimal number of variables randomly sampled as candidates at each split (`mtry`). FALSE is the default due to potentially extensive computation times.
- `set_seed`: seed for reproducibility, defaults to 42
- `ntree`: integer, number of trees (defaults to 1000 trees)
- `n_cores`: integer, if >1, computations will be parallelized across the amount of cores specified in `n_cores`. This is important especially for larger datasets as the random forest training can be very time-consuming if done sequentially.

1.2.5 Group 3 diagnostics

To evaluate the association of the misssing indicator variable of a POC (M_{POC}) and the studied outcome in Group 3 Diagnostics, conventional univariate and multivariate regression models are employed (latter of which are adjusted for other covariates X_j). The type of regression

model needs to be chosen using the `model` parameter and is determined by the type of outcome that is being studied. Users can choose from multiple possible outcome models, including:

- Linear regression [23] for continuous outcomes (Equation 5)
- Cox proportional hazards regression [24] for time-to-event outcomes (Equation 6).
- Generalized linear models (e.g., logistic regression [25], Equation 7) for which the family of conditional distributions of the outcome variable can be selected using the `glm_family` parameter and which needs to be an object of type `stats::family`. Possible options are:
  - binomial(link = "logit")
  - gaussian(link = "identity")
  - Gamma(link = "inverse")
  - inverse.gaussian(link = "1/mu^2")
  - poisson(link = "log")
  - quasi(link = "identity", variance = "constant")
  - quasibinomial(link = "logit")
  - quasipoisson(link = "log")

The `form_lhs` specifies the left-hand side of the outcome formula, which, in case of `model = "linear"` or `model = "glm"`, just reflects the name of the column that contains the outcome and the form `Surv(time, status)` for `model = "cox"` indicating the time-to-event variable and the event status (0/1). The output of the functions automatically returns results for both univariate and multivariate models and a user has the choice to exponentiate the $\beta_1 M_{POC}$ estimate if desired using the `exponentiated` parameter.

$$Y = \beta_0 + \beta_1 M_{POC} + ... + \beta_2 X_j \tag{5}$$

$$h(t) = h_0(t) e^{\sum \beta_1 M_{POC} + ... + \beta_2 X_j} \tag{6}$$

$$\ln(\frac{p}{p-1}) = \beta_0 + \beta_1 M_{POC} + ... + \beta_2 X_j \tag{7}$$

References

1 Weberpals J, Raman SR, Shaw PA, *et al.* A principled approach to characterize and analyze partially observed confounder data from electronic health records. *Submitted*. 2023.

2 Moreno-Betancur M, Lee KJ, Leacy FP, *et al.* Canonical Causal Diagrams to Guide the Treatment of Missing Data in Epidemiologic Studies. *American Journal of Epidemiology*. 2018;187:2705–15.

3 Sondhi A, Weberpals J, Yerram P, *et al.* A systematic approach towards missing lab data in electronic health records: A case study in non-small cell lung cancer and multiple myeloma. *CPT: Pharmacometrics & Systems Pharmacology*. Published Online First: 15 June 2023. doi: 10.1002/psp4.12998

4 Iannone R, Cheng J, Schloerke B, *et al.* Gt: Easily create presentation-ready display tables. 2023. https://CRAN.R-project.org/package=gt

5 Wickham H. ggplot2: Elegant graphics for data analysis. 2016. https://ggplot2.tidyverse.org

6 Tierney N, Cook D. Expanding tidy data principles to facilitate missing data exploration, visualization and assessment of imputations. 2023;105. doi: 10.18637/jss.v105.i07

7 Buuren S van, Groothuis-Oudshoorn K. Mice: Multivariate imputation by chained equations in r. *Journal of Statistical Software*. 2011;45:1–67.

8 Ruddle RA, Adnan M, Hall M. Using set visualisation to find and explain patterns of missing values: a case study with NHS hospital episode statistics data. *BMJ Open*. 2022;12:e064887.

9 Hotelling H. The Generalization of Student's Ratio. *The Annals of Mathematical Statistics*. 1931;2:360–78.

10 Curran J, Hersh T. Hotelling: Hotelling's t^2 test and variants. 2021. https://CRAN.R-project.org/package=Hotelling

11 Multivariate analysis of means for two groups. https://rpubs.com/juanhklopper/multivariate_comparison_of_means_of_two_groups (accessed 19 November 2023)

12 Applied multivariate statistical analysis (PennState). https://online.stat.psu.edu/stat505/lesson/7/7.1/7.1.15 (accessed 19 November 2023)

13 Little RJA. A Test of Missing Completely at Random for Multivariate Data with Missing Values. *Journal of the American Statistical Association*. 1988;83:1198–202.

14 Enders CK. *Applied missing data analysis*. Guilford Publications 2022.

15 Little's missing completely at random (MCAR) test. https://search.r-project.org/CRAN/refmans/misty/html/na.test.html (accessed 19 November 2023)

16 Austin PC. An Introduction to Propensity Score Methods for Reducing the Effects of Confounding in Observational Studies. *Multivariate Behavioral Research*. 2011;46:399–424.

17 Normand S-LT, Landrum MB, Guadagnoli E, *et al.* Validating recommendations for coronary angiography following acute myocardial infarction in the elderly. *Journal of Clinical Epidemiology*. 2001;54:387–98.

18 Yoshida K, Bartel A. Tableone: Create 'table 1' to describe baseline characteristics with or without propensity score weights. 2022. https://CRAN.R-project.org/package=tableone

19 Liaw A, Wiener M. Classification and regression by randomForest. 2002;2:18–22. https://CRAN.R-project.org/doc/Rnews/

20 Shwartz-Ziv R, Armon A. Tabular data: Deep learning is not all you need. *Information Fusion*. 2022;81:84–90.

21 Grinsztajn L, Oyallon E, Varoquaux G. Why do tree-based models still outperform deep learning on tabular data? 2022. https://arxiv.org/abs/2207.08815

22 Beaulieu-Jones BK, Lavage DR, Snyder JW, *et al.* Characterizing and Managing Missing Structured Data in Electronic Health Records: Data Analysis. *JMIR Medical Informatics*. 2018;6:e11.

23 R Core Team. R: A language and environment for statistical computing. 2022. https://www.R-project.org/

24 Therneau TM. A package for survival analysis in r. 2023. https://CRAN.R-project.org/package=survival

25 Friedman J, Tibshirani R, Hastie T. Regularization paths for generalized linear models via coordinate descent. 2010;33. doi: 10.18637/jss.v033.i01
